# Grammatical Parallelism in Aphasia: A Lesion-Symptom Mapping Study

**DOI:** 10.1162/nol_a_00117

**Published:** 2023-10-31

**Authors:** William Matchin, Dirk-Bart den Ouden, Alexandra Basilakos, Brielle Caserta Stark, Julius Fridriksson, Gregory Hickok

**Affiliations:** Department of Communication Sciences and Disorders, University of South Carolina, Columbia, SC, USA; Department of Speech, Language and Hearing Sciences, Program for Neuroscience, Indiana University Bloomington, Bloomington, IN, USA; Department of Cognitive Sciences, Department of Language Science, University of California, Irvine, Irvine, CA, USA

**Keywords:** syntax, aphasia, agrammatism, paragrammatism, lesion-symptom mapping

## Abstract

Sentence structure, or syntax, is potentially a uniquely creative aspect of the human mind. Neuropsychological experiments in the 1970s suggested parallel syntactic production and comprehension deficits in agrammatic Broca’s aphasia, thought to result from damage to syntactic mechanisms in Broca’s area in the left frontal lobe. This hypothesis was sometimes termed *overarching agrammatism*, converging with developments in linguistic theory concerning central syntactic mechanisms supporting language production and comprehension. However, the evidence supporting an association among receptive syntactic deficits, expressive agrammatism, and damage to frontal cortex is equivocal. In addition, the relationship among a distinct grammatical production deficit in aphasia, paragrammatism, and receptive syntax has not been assessed. We used lesion-symptom mapping in three partially overlapping groups of left-hemisphere stroke patients to investigate these issues: grammatical production deficits in a primary group of 53 subjects and syntactic comprehension in larger sample sizes (*N* = 130, 218) that overlapped with the primary group. Paragrammatic production deficits were significantly associated with multiple analyses of syntactic comprehension, particularly when incorporating lesion volume as a covariate, but agrammatic production deficits were not. The lesion correlates of impaired performance of syntactic comprehension were significantly associated with damage to temporal lobe regions, which were also implicated in paragrammatism, but not with the inferior and middle frontal regions implicated in expressive agrammatism. Our results provide strong evidence against the overarching agrammatism hypothesis. By contrast, our results suggest the possibility of an alternative grammatical parallelism hypothesis rooted in paragrammatism and a central syntactic system in the posterior temporal lobe.

## INTRODUCTION

### Agrammatism and Syntactic Parallelism in Aphasia

Syntax, or the ability to combine words into hierarchical structures, enables a core component of human linguistic creativity: the ability to make novel sentences of unbounded size and number (Chomsky, 1965; von Humboldt, 1836). The nature of syntactic deficits in aphasia and the role of Broca’s area in such deficits has a long and complicated history. From the beginning of the 20th century until the 1970s, agrammatism in people with nonfluent Broca’s aphasia was defined as the systematic reduction of syntactic complexity and omission of functional elements (such as auxiliary verbs and articles) in speech production; syntactic comprehension was assumed to be intact (Forster, 1919; Isserlin, 1922). This was consistent with the classical model of language in the brain espoused by Wernicke, Lichtheim, and later Geschwind, positing that frontal damage, which was linked to Broca’s aphasia, produced expressive language deficits in the absence of notable receptive deficits (Geschwind, 1970, 1972, 1979; Lichtheim, 1885; Wernicke, 1874).

The received view of impaired production but preserved comprehension in agrammatism was questioned in the 1970s, when some studies (typically with small numbers of participants and an absence of detailed lesion analysis) revealed apparent syntactic comprehension deficits in people with agrammatic Broca’s aphasia (Caramazza & Zurif, 1976; Zurif et al., 1972; patients with conduction aphasia, without agrammatic production deficits, showed the same comprehension pattern, a fact rarely discussed in current literature). A highly influential study by Caramazza and Zurif (1976) assessed the syntactic comprehension abilities of English-speaking people with agrammatic Broca’s aphasia using semantically reversible sentences with noncanonical word order. In both (1) and (2), below, the sentences have a noncanonical word order, in which the initial noun phrase and participant of the main clause, the man in (1) and the apple in (2), are the object of the verbs in the embedded clauses. Reversible sentences consisted of those in which the doer and receiver of the action could switch positions while the sentence still made sense (1), as opposed to semantically constrained sentences which would be thematically impossible if the doer and receiver were switched (2).

1. The man that the woman is hugging is happy (reversible, noncanonical word order, **poor performance**)
2. The apple that the boy is eating is red (semantically constrained, noncanonical word order, **good performance**)

This study showed that comprehension of *semantically constrained* sentences with noncanonical word order (2) was good, but that comprehension of *reversible* sentences with noncanonical word order (1) was poor. Caramazza and Zurif (1976) argued that people with agrammatic Broca’s aphasia lacked normal syntactic ability not only for production but also for comprehension, explaining the selective pattern of comprehension deficits. This pattern could be explained via the preservation of word-level understanding and interpretive heuristics based on semantic plausibility and assuming the first noun to be the agent of the action.

Such results shifted the standard assumption in aphasiology toward *syntactic parallelism*: the idea that syntactic comprehension deficits accompanied syntactic production deficits in agrammatic Broca’s aphasia (Berndt & Caramazza, 1980; Kean, 1977; Saffran et al., 1980; Schwartz et al., 1980; Zurif, 1980). The syntactic parallelism hypothesis was sometimes termed *overarching agrammatism* (Grodzinsky, 2000a; Swinney & Zurif, 1995), emphasizing that the comprehension and production deficits in agrammatism resulted from disruption to a common underlying central syntactic mechanism supporting both the production and comprehension of language (Chomsky, 1965, 1980). This represented a major shift in thinking regarding aphasiology and the neurobiology of language. Instead of talking about language deficits and neurological models in terms of production or comprehension functions, the vocabulary of linguistic theory regarding central functions of syntax (assumed to be localized to Broca’s area, or the left posterior inferior frontal gyrus) and semantics (assumed to be localized to left posterior temporal cortex) was used to define the language–brain relationship (see also Jakobson, 1956, and Jakobson & Halle, 1956, for early views on the application of linguistics to aphasiology). This was in part based on the assumed relationship between Broca’s aphasia and Broca’s area, although we note that Broca’s aphasia may be caused by lesions not involving Broca’s area (Fridriksson et al., 2007), and damage to Broca’s area alone is insufficient to cause Broca’s aphasia (Fridriksson et al., 2015; Gajardo-Vidal et al., 2021; Mohr et al., 1978).

The seemingly tight convergence between linguistic theory and aphasiology that this work pointed toward began to unravel with demonstrations of intact receptive syntactic abilities in people with agrammatic Broca’s aphasia in the 1980s. Specifically, syntactic acceptability judgments, subtle and direct tests of syntactic ability, were shown to be mostly intact in people with Broca’s aphasia and expressive agrammatism (Linebarger et al., 1983; Wilson & Saygın, 2004; Wulfeck & Bates, 1991). These authors proposed instead that the relationship between the syntactic mechanisms identified in linguistic theory and language deficits in aphasia was much more indirect, with agrammatic comprehension deficits in aphasia reflecting the impaired ability to make use of intact syntactic representations for semantic interpretation. Some authors sought to preserve aspects of the overarching agrammatism hypothesis by positing a more restricted syntactic deficit, one tied to mechanisms devoted to the movement of sentence elements (Chomsky, 1981) that explained difficulties with noncanonical sentences (Grodzinsky, 1986; Hickok et al., 1993). However, by the 1990s, many researchers in aphasiology had abandoned the syntactic parallelism hypothesis (Goodglass et al., 1993; Hickok & Avrutin, 1995; Kean, 1995; Swinney & Zurif, 1995; for a review, see Matchin & Rogalsky, 2023).

Despite this, the hypothesis of overarching agrammatism never fully disappeared, and many researchers have continued to focus on nonfluent aphasia patients, primarily those with Broca’s aphasia and/or expressive agrammatism, and have continued to use noncanonical sentence structures as a diagnostic tool for receptive syntax (Cho-Reyes & Thompson, 2012; Grodzinsky, 2000b; Grodzinsky & Finkel, 1998; Mesulam et al., 2015; Santi & Grodzinsky, 2007; Thompson et al., 1997; Thompson et al., 2013; Thompson & Shapiro, 2005; Tyler et al., 2011; Wilson, Dronkers, et al., 2010). In addition, the arrival of functional neuroimaging in the 1990s breathed new life into the overarching agrammatism hypothesis, with several studies of syntactic comprehension showing effects in Broca’s area (Dapretto & Bookheimer, 1999; Embick et al., 2000; Friederici et al., 2000; Moro et al., 2001; Stromswold et al., 1996). Finally, research on the neural basis of word-level processes (lexical access) demonstrated associations within the left posterior temporal lobe (Dronkers et al., 2004; Hickok & Poeppel, 2000; Lau et al., 2008; Levelt, 2001; Levelt et al., 1999; Pinker & Ullman, 2002). Together, these findings reinforced a framework for language in the brain that posited a syntactic computation function in Broca’s area and a lexical storage function in left posterior temporal cortex (Friederici, 2002; Hagoort, 2005), aligning two core aspects of language with the two classical language regions. Thus, despite striking contradictory evidence from aphasiology, the overarching agrammatism hypothesis and the concept of a central syntactic hub in Broca’s area continues to have substantial influence in psychology, linguistics, and neuroscience (Arbib, 2016; Bozic et al., 2015; D’Ausilio et al., 2012; Friederici, 2017; Hagoort, 2013, 2016; Hagoort & Indefrey, 2014; Kuperberg, 2007; Menenti et al., 2011; Momma & Phillips, 2018; Ocampo & Kritikos, 2011; Pulvermüller & Fadiga, 2010; Rilling, 2014; Tyler et al., 2010; Wilson, Dronkers, et al., 2010).

### Paragrammatism

Agrammatism is not the only grammatical production deficit in aphasia. In the early 1900s, clinicians described an expressive disorder termed *paragrammatism*, which is associated with fluent aphasia and characterized by grammatical distortion but without the overall reduction/simplification that is characteristic of agrammatism (Bonhoeffer, 1902; Butterworth & Howard, 1987; Goodglass et al., 1993; Heeschen, 1985; Kleist, 1914). This disorder has received vanishingly little attention in the last decades. In recent work, Matchin et al. (2020) found a double dissociation in the lesion distributions associated with agrammatism versus paragrammatism: Agrammatism was associated with inferior and middle frontal damage including Broca’s area, but not left temporal cortex, consistent with several previous studies (den Ouden et al., 2019; Sapolsky et al., 2010; Wilson et al., 2011; Wilson, Henry, et al., 2010). By contrast, paragrammatism was associated with damage to the posterior temporal cortex, but not frontal cortex, consistent with case studies (Yagata et al., 2017). Recent lesion-symptom mapping (LSM) and connectome-based LSM studies have identified a robust association between syntactic comprehension deficits and damage to the middle posterior temporal lobe, with minimal or no implication of damage to or disconnection of Broca’s area, assessed via noncanonical sentence comprehension (Billot et al., 2022; den Ouden et al., 2019; Dronkers et al., 2004; Kristinsson et al., 2020; Lukic et al., 2021; Lwi et al., 2021; Matchin, Basilakos, et al., 2022; Matchin, den Ouden, et al., 2022; Rogalsky et al., 2018), syntactic acceptability judgments (Wilson & Saygın, 2004), and sentence comprehension with word production ability as a covariate (Pillay et al., 2017; cf. Adezati et al., 2022, which found that sentence comprehension deficits were associated with damage to both regions). This suggests that parallel grammatical deficits in aphasia (rooted in paragrammatism, rather than agrammatism) may result from common injury to the posterior temporal lobe. Indeed, some studies assessing syntactic production deficits measured using the Northwestern Assessment of Verbs and Sentences (NAVS) sentence priming production task have found that superior temporal lobe damage, and not frontal damage, is associated with production deficits (den Ouden et al., 2019; Lukic et al., 2021), although we note that this test critically involves correct comprehension to perform the task, conflating these results with comprehension ability.

### Hypotheses and Predictions

Recent functional neuroimaging studies often find syntax-related activations in the inferior frontal (typically along with the posterior temporal) lobe (Blank et al., 2016; Fedorenko et al., 2016; Matchin, Liao, et al., 2019; Nelson et al., 2017; Zaccarella, Meyer, et al., 2017; cf. several recent studies that highlight temporal and not frontal lobe, Brennan et al., 2016; Flick & Pylkkänen, 2020; Matar et al., 2021; Matchin, Brodbeck, et al., 2019; Matchin, İlkbaşaran, et al., 2022; Murphy et al., 2022; Stanojević et al., 2023). While these studies have long suggested to researchers that frontal lobe structures support syntactic processing, the correlational nature of functional imaging precludes an assessment of the extent to which these structures are causally implicated. Lesion studies, by contrast, can provide critical evidence regarding the causal role of these regions (Rorden & Karnath, 2004). Accordingly, many of the aphasiological studies reviewed above have concluded that frontal structures are causally implicated in receptive syntax, in accordance with the “overarching agrammatism” hypothesis. However, this question of syntactic parallelism, rooted in agrammatism, has not been revisited and comprehensively addressed using modern LSM methods (cf. den Ouden et al., 2019; Lukic et al., 2021, which assessed both production and comprehension and only found temporal lobe damage associated with objective quantitative assessments. Den Ouden et al. found that frontal damage was associated with expressive agrammatism as identified with perceptual analysis, but did analyze the relationship of these categorizations with syntactic comprehension variables). Nor has a syntactic parallelism hypothesis rooted in paragrammatism been investigated.

Here we systematically (re)assess these issues in three partially overlapping groups of people with post-stroke aphasia. In order for syntactic parallelism to hold, the association between syntactic comprehension and production deficits should be robust to various measures and analyses. Thus, we assessed syntactic comprehension ability in a variety of ways. In the literature, different approaches have been used, with some authors using sentence comprehension measures that implicate syntactic abilities that minimize lexical demands (Dronkers et al., 2004; Gorno-Tempini et al., 2004; Matchin, den Ouden, et al., 2022; Mesulam et al., 2015; Thompson et al., 2013; Wilson, Dronkers, et al., 2010), and others combining such measures with covariates in order to more purely isolate syntactic abilities (Kristinsson et al., 2020; Magnusdottir et al., 2013; Matchin, Basilakos, et al., 2022; Pillay et al., 2017; Rogalsky et al., 2018). We adopted both approaches here, using two different sentence comprehension measures to assess receptive syntax: the Sequential Commands subtest of the Western Aphasia Battery—Revised (WAB-R; Kertesz, 2006) and the comprehension of noncanonical sentence structures (Cho-Reyes & Thompson, 2012; Magnusdottir, 2005), each with or without a covariate to attempt to control for lexical-semantic processing, for a total of four analyses of syntactic comprehension. The overarching agrammatism hypothesis predicts that both expressive agrammatism and syntactic comprehension deficits follow from lesions to the same parts of the frontal lobe. The syntactic parallelism hypothesis rooted in paragrammatism predicts that both expressive paragrammatism and syntactic comprehension deficits will follow from lesions to the same parts of the posterior temporal lobe. We expected that our results would speak against the overarching agrammatism view and instead support the overarching paragrammatism view, given previous suggestive neuropsychological data of intact syntactic comprehension in agrammatism and previous associations of syntactic comprehension deficits with posterior temporal lobe lesions.

## MATERIALS AND METHODS

### Participants

Participants were drawn from a database of individuals with chronic, post-stroke aphasia who have completed testing for various studies conducted at the University of South Carolina and the Medical University of South Carolina over the last 15 years. All were native speakers of English, had suffered an ischemic stroke to the left hemisphere at least six months prior to the study, and presented with language difficulties (most participants were classified as aphasic according to the WAB-R [Kertesz, 2006]; however, some scored outside of the aphasic range by the time of examination. All participants had presented with aphasia in the acute phase following their stroke, which formed the basis of their enrollment in the study). We performed retrospective analyses in three overlapping groups of stroke survivors. Group 1 consisted of 53 participants (all of whom were included in Group 3 and 42 of whom were included in Group 2) who were perceptually assessed for agrammatism and paragrammatism. Group 2 consisted of a subset of 130 participants who were assessed on one of two similar tests of sentence comprehension involving canonical and noncanonical sentence structures. Group 3 consisted of 218 participants who were assessed on the WAB-R. Table 1 provides demographic, lesion volume, and aphasia severity for each of the three groups (demographic information broken down by perceptual ratings of agrammatism and paragrammatism can be found in Table 1 of Matchin et al., 2020). All procedures were approved by the internal review boards at each institution and informed consent was obtained.

**Table 1.** Participant information for the three partially overlapping groups of participants.

	Group 1	Group 2	Group 3
Tasks	Perceptual ratings of agrammatism and paragrammatism	Noncanonical sentence comprehension	Western Aphasia Battery—Revised (WAB-R)
Number of participants in group	53	130 (42 participants from Group 1 included)	218 (all participants from Groups 1 and 2 included)
Sex	35 male, 18 female	83 male, 47 female	133 male, 85 female
Pre-stroke handedness	45 right-handed, 2 left-handed, 6 unknown	108 right-handed, 8 left-handed, 14 unknown	178 right-handed, 9 left-handed, 31 unknown
Mean age at testing (years)	58.9 (*SD* = 12.2)	60.0 (*SD* = 10.7)	60.0 (*SD* = 11.4)
Mean months post-stroke at initial testing	48.6 (*SD* = 53.5)	45.3 (*SD* = 50.4)	43.0 (*SD* = 48.4)
Mean education (years)	15.8 (*SD* = 2.3)	15.4 (*SD* = 2.4), *N = 128	15.0 (*SD* = 2.3), *N = 210
Mean lesion volume (mm^3^)	125,102 (*SD* = 85,490)	111,267 (*SD* = 92,645)	120,855 (*SD* = 97,488)
Mean WAB-R AQ	68.2 (*SD* = 16.7)	65.3 (*SD* = 26.9)	61.4 (*SD* = 28.1)

*Note*. *SD* = standard deviation. AQ = aphasia quotient of the WAB-R, a summary measure of overall language ability, with 0 being the most severe score. The WAB-R denotes that an AQ of <93.8 denotes presence of clinical aphasia.

* Education information was not available for all participants, the number for which education information indicated here.

### Measures and Procedure

All 218 participants were evaluated using the WAB-R (Kertesz, 2006) to determine the presence and severity of aphasia. The test was administered and scored by certified speech-language pathologists with extensive experience evaluating individuals with aphasia. The WAB-R contains multiple subtests to evaluate production and comprehension ability. Here we focus on the Sequential Commands and the Auditory Word Recognition subtests. The Sequential Commands subtest consists of 11 sentential instructions for actions to perform. It requires the participant to process basic syntactic relations indicated with prepositional phrase modifiers and connectives, while minimizing lexical demands (using repeated high frequency nouns referring to common objects located in the testing area). A previous study also used the Sequential Commands subtest of the WAB-R to assess syntactic comprehension (Gorno-Tempini et al., 2004), but did so in conjunction with additional measures including complex sentences with noncanonical word order. While some of the commands can be performed correctly without syntactic analysis, relying on lexical comprehension alone (e.g., *raise your hand*), the bulk of the total score requires analyzing both the lexical items and their syntactic arrangement to perform correctly (e.g., *point to the comb with the pen*). Indeed, Schwartz et al. (1980) report that people with agrammatic Broca’s aphasia have difficulty comprehending sentences of this type (recall that having Broca’s aphasia does not necessarily imply that damage to Broca’s area is the cause of the deficits, Fridriksson et al., 2015). Participants can receive partial credit for correctly performing a subset of the actions indicated in a command; full credit required performing all of the indicated actions in the correct order. We used raw scores on this subtest for our sequential commands measure.

To control for lexical-semantic processing, in additional analyses we incorporated the Auditory Word Recognition subtest as a covariate with the sequential commands measure to produce the sequential commands_audwords_ analysis. This subtest involves asking the participant to point to real-world objects or printed images as requested. Some of these objects are contained within the Sequential Commands subtest. Participants are prompted with a sentence, for example, “point to the __” or “show me the __.” The test involves multiple types of tested words, including real household objects (e.g., cup, comb), pictured objects (the same as real objects), pictured shapes (e.g., square, circle), pictured letters (e.g., J, F), pictured numbers (e.g., 5, 61), pictured colors (e.g., blue, red), real world furniture (e.g., window, chair), real world body parts (e.g., ear, nose), real world fingers (e.g., thumb, index finger), and real world body parts on the correct side (e.g., right ear, left knee). For each item the participant receives 1 point, for a total of 60 points. Thus, for our sequential commands_audwords_ analysis we assessed sequential commands incorporating the Auditory Word Recognition subtest as a covariate.

We included two additional analyses of syntactic comprehension, noncanonical and noncanonical_active_, based on the standard approach to syntactic comprehension in aphasia centered around noncanonical sentence structures introduced by Caramazza and Zurif (1976) and developed since then by many authors (Caplan et al., 1996; Cho-Reyes & Thompson, 2012; Dronkers et al., 2004; Magnusdottir, 2005; Magnusdottir et al., 2013). We used data that have been previously reported in LSM analyses of these tasks in 130 people with chronic stoke-based aphasia, one study reporting the lesion correlates of noncanonical sentence comprehension combined with active sentence comprehension as a covariate (Matchin, Basilakos, et al., 2022), and one study reporting the lesion correlates of noncanonical sentence comprehension by itself (Matchin, den Ouden, et al., 2022). Some of these same participants have also been reported in another study (den Ouden et al., 2019).

The noncanonical measure was derived from the performance on noncanonical structures from the NAVS (82 participants; Cho-Reyes & Thompson, 2012) or the Test of Syntax (ToS; 48 participants; Magnusdottir, 2005; Magnusdottir et al., 2013). The NAVS involves testing the comprehension of a variety of canonical and noncanonical sentence types, each with five total trials, assessed via pointing to the correct picture. The performance on the three noncanonical sentence types of the NAVS combined has been used as an index of syntactic comprehension: passives (with a by-phrase) e.g., *the dog is chased by the cat*, object-extracted WH-questions, e.g., *who is the cat chasing?*, and object-relatives, e.g., *Pete saw the boy who the girl is pulling*, for a maximum score of 15 (den Ouden et al., 2019; Mesulam et al., 2015; Thompson et al., 2013), a procedure similar to that used with other tests. The ToS (Magnusdottir, 2005; Magnusdottir et al., 2013) includes a similar set of canonical and noncanonical sentence types, each with five total trials: passives (with a by-phrase), for example, “The boy is painted by the girl”; object-extracted WH- questions, for example, “Which boy is the girl painting?”; and object clefts, for example, “It is the girl that the boy paints.” The sentence types across the two tasks are not strictly identical, but involve essentially the same structures with the same degree of complexity, including the key factor of noncanonical object-first word order. Therefore, for participants who were not assessed with the NAVS, we calculated the equivalent scores on the ToS (correct noncanonical trials, out of 15 points). This score, correct noncanonical trials out of 15, comprised the noncanonical measure. The noncanonical_active_ analysis was derived by performing a linear regression on the noncanonical score, including performance on simple active sentences as a covariate (active sentence comprehension, out of 5 points), which was included in both the NAVS and ToS, to control for lexical-semantic processing.

### Agrammatism/Paragrammatism Ratings

For our measures of syntactic production deficits, agrammatism and paragrammatism, we used the data from 53 participants reported in Matchin et al. (2020): categorical perceptual ratings, formed as a consensus of four expert raters based on the patients’ unconstrained retelling of the Cinderella Story in their own words, following the AphasiaBank protocol (MacWhinney et al., 2011). Full details of participant selection, testing, and evaluation are provided in Matchin et al. (2020). Briefly, 100 people with chronic stroke-based aphasia, who partook in a broader study of aphasia recovery, were recorded as they performed the Cinderella Story retelling task from the AphasiaBank protocol (MacWhinney et al., 2011). Recordings ranged from a few seconds to several minutes. Four expert raters, blind to any information about each participant other than their recording, watched the recordings as many times as needed and rated each participant as agrammatic (11 participants), paragrammatic (21 participants), both agrammatic and paragrammatic (4 participants), or no grammatical deficit (17 participants). Following this, a discussion was held among all four raters to develop a single consensus rating for each participant. 47 participants were excluded due to severely limited speech output or unintelligibility (45 participants) and poor audio recording quality (2 participants). The four patients who were identified as exhibiting some features of both classifications were included in analyses of both of these measures; thus, we had a total of 15 agrammatic participants and 25 paragrammatic participants. Only 42 of these original 53 participants also performed the canonical/noncanonical sentence comprehension tasks, thus the numbers for each subgroup are as follows: agrammatic (7 participants), paragrammatic (16 participants), no grammatical deficit (15 participants), or both agrammatic and paragrammatic (4 participants). Throughout the paper, we express the general concepts discussed in the literature of agrammatism (and agrammatic speech) and paragrammatism (and paragrammatic speech) using regular typeface. We express the corresponding perceptual classification of these concepts as applied to our participant groups using small capital typeface, that is, agrammatism/agrammatic and paragrammatism/paragrammatic, and correspondingly, not-agrammatic and not-paragrammatic for patients that were classified as not having these grammatical production deficits.

### Neuroimaging and Lesion Mapping

High-resolution magnetic resonance imaging (MRI) data (T1- and T2-weighted images) were collected at University of South Carolina and the Medical University of South Carolina on a 3T Siemens Trio scanner with a 12-element head coil. T1-weighted MRI images were collected using an MP-RAGE sequence, voxel dimensions 1 mm^3^, 256 × 256 matrix, 9° flip angle, TR 2,250 ms, either 160 slices with inversion time of 900 ms and echo time of 4.52 ms, or 192 slices with inversion time of 925 ms and TE of 4.15 ms with parallel imaging (GRAPPA = 2, 80 reference lines). T2-weighted MRI images were collected using a sampling perfection with application optimized contrasts with a different flip angle evolution sequence (3D-SPACE). This 3D turbo spin echo scan has 192 slices 1 mm thick, TR of 2,800 ms, TE of 402 ms, variable flip angle, 256 × 256 matrix, with parallel imaging (GRAPPA = 2, 120 reference lines).

**Figure 1.**
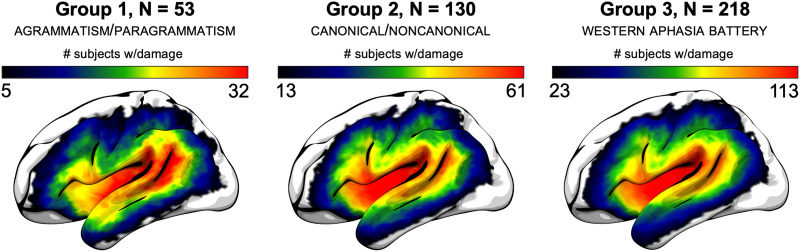
Lesion overlap maps for the three groups of participants detailed in Table 1. The lower bound indicates the lower bound of the lesion load threshold, i.e. the minimum number of participants with damage to a voxel required for statistical analysis; the upper bound indicates maximum overlap. (Left) The primary group (Group 1) of 53 participants who were assessed for agrammatism and paragrammatism. (Middle) The group of 130 participants who were assessed for Noncanonical sentence comprehension (Group 2), including 42 of the participants from Group 1. (Right) The broader set of 218 participants who were enrolled in all measures (Group 3).

Lesions were demarcated onto each participant’s T2 image by an expert neurologist (Dr. Leonardo Bonilha) or an expert cognitive neuroscientist (Dr. Roger Newman-Norlund) extensively trained by Dr. Bonilha (with consultation as needed with an expert on lesion mapping, Dr. Chris Rorden), both blind to the behavioral data. Lesion maps were then aligned to the high resolution T1 image. Lesions were replaced with the corresponding brain structure from the intact hemisphere, and this image as well as the lesion map in participant space were subsequently warped to Montreal Neurological Institute (MNI) space (Nachev et al., 2008) using SPM12 (Ashburner & Friston, 2005). The warped lesion map was then binarized with a 50% probability threshold, which was used to perform voxel-wise and region of interest (ROI) analyses. Figure 1 shows lesion overlap maps for each of the three groups indicated in Table 1, indicating the distribution of lesions and lesion coverage.

### Behavioral Analyses

In our first pass of behavioral analyses, we compared agrammatic and paragrammatic participants to their not-agrammatic and not-paragrammatic counterparts on each analysis of syntactic comprehension using linear regression in JASP Team (2023). We performed these analyses without lesion volume as a covariate in order to replicate previous studies that have found associations between agrammatic Broca’s aphasia and syntactic comprehension deficits that did not address lesion volume as a confounding variable. We corrected for multiple comparisons using a Bonferroni correction with an adjusted alpha threshold of *p* < 0.025 for the four comparisons within each family of tests (treating agrammatism and paragrammatism as separate families), using one-sided tests (negative associations only), controlling the total family-wise error at *p* < 0.05.

In a second set of behavioral analyses, we incorporated lesion volume as a covariate in linear regression. agrammatic participants in our sample have nearly twice the lesion volume of the paragrammatic participants (Matchin et al., 2020), thus presenting potential confounds. The lesion volume differences are likely due to vasculature differences in these lesion distributions (DeMarco & Turkeltaub, 2018). In particular, we suspected that some reports of syntactic comprehension deficits in agrammatic patients might be due to their (comparatively larger) frontal-based lesions encroaching into the temporal lobe, which is consistent with the fact that chronic Broca’s aphasia reliably implicates posterior temporal as well as frontal damage (Fridriksson et al., 2015). By incorporating lesion volume as a covariate into our analyses, we controlled for this potentially confounding factor. We corrected for multiple comparisons using a Bonferroni correction with an adjusted alpha threshold of *p* < 0.025 for the four comparisons within each family of tests (treating agrammatism and paragrammatism as separate families), (negative associations only), controlling the total family-wise error at *p* < 0.05.

We also report supplementary analyses in parallel with those described above, incorporating age at testing, years of education, and WAB-R AQ as covariates (see Supporting Information, available at https://doi.org/10.1162/nol_a_00117, for details), given that these variables likely relate to better outcomes (Lwi et al., 2021).

### Lesion Analyses

We performed two types of lesion-behavior analyses. First, we ran an exploratory whole-brain analysis at the voxel level, reporting unthresholded results to provide an overall picture of the lesion distributions associated with all behavioral measures (agrammatism, paragrammatism, auditory word recognition, sequential commands, sequential commands_audwords_, active sentence comprehension, noncanonical, noncanonical_active_). Then, we performed an atlas-based analysis using the parcellation developed by Faria et al. (2012), which contains both gray and white matter regions, in NiiStat (NITRC, 2020) to identify regions significantly associated with agrammatism and paragrammatism, correcting for multiple comparisons using permutation testing (10,000 permutations). For all of these analyses, to ensure reliable localization we used a lesion load threshold of 10% of sample (22 participants for sequential commands and sequential commands_audwords_, 13 participants for noncanonical and noncanonical_active_, and 5 participants for agrammatism and paragrammatism), and lesion volume was included as a covariate in all analyses (DeMarco & Turkeltaub, 2018; Ivanova et al., 2021).

Second, we assessed the extent to which damage to the regions implicated in agrammatism and paragrammatism were associated with each of the four syntactic comprehension analyses. We first created ROIs based on the significant results from the atlas-based analyses described above, by combining the significant regions together. Thus, for agrammatism, the ROI consisted of the combination of the inferior frontal gyrus, pars opercularis, the inferior frontal gyrus, pars triangularis, and the posterior middle frontal gyrus, whereas for paragrammatism, the ROI consisted of the middle superior temporal gyrus, posterior superior temporal gyrus, and posterior middle temporal gyrus. We then used these lesion distributions as ROIs for further analysis of the four syntactic comprehension analyses. We first calculated proportion damage to each ROI for each participant. We then adjusted the data using a rationalized arcsine transform in order to deal with nonnormality and unequal variances associated with proportional data (Studebaker, 1985). We then performed one-sided linear regression analyses relating the damage values for each ROI and each behavioral analysis, separately, incorporating lesion volume as a covariate. For the analyses of sequential commands_audwords_ and noncanonical_active_, we analyzed sequential commands and noncanonical, incorporating the additional covariates of Auditory Word Comprehension and Active Sentence Comprehension, respectively. We corrected for multiple comparisons reflecting both the one-sided tests (negative associations only) and a Bonferroni correction with an adjusted alpha threshold of *p* < 0.025 for each family of tests (treating agrammatism and paragrammatism as separate families), controlling the total error at *p* < 0.05.

Finally, to illustrate concretely the fact that subjects with expressive agrammatism also tend to have large lesions encroaching on the temporal lobe, we also computed a lesion overlap map of subjects with expressive agrammatism who also showed the classic agrammatic comprehension profile, numerically worse performance on noncanonical relative to canonical structures (seven subjects). That is, we identified all subjects who performed worse by at least one trial on the three noncanonical conditions relative to the three canonical conditions, and then summed the lesion maps for all seven of these subjects, revealing the extent of lesion overlap in each region within this group. We supplemented this with additional LSM analyses of expressive agrammatism, with and without a lesion volume covariate (replicating figures presented in Matchin et al., 2020).

## RESULTS

### Behavioral Data (No Lesion Volume Covariate)

We compared each group of grammatically impaired participants (agrammatic, paragrammatic) to their *not* grammatically impaired counterparts (not-agrammatic, not-paragrammatic) with respect to our four syntactic comprehension analyses: sequential commands, sequential commands_audwords_ (incorporating the auditory word recognition covariate), noncanonical, noncanonical_active_ (incorporating the active sentence comprehension covariate).

**Figure 2.**
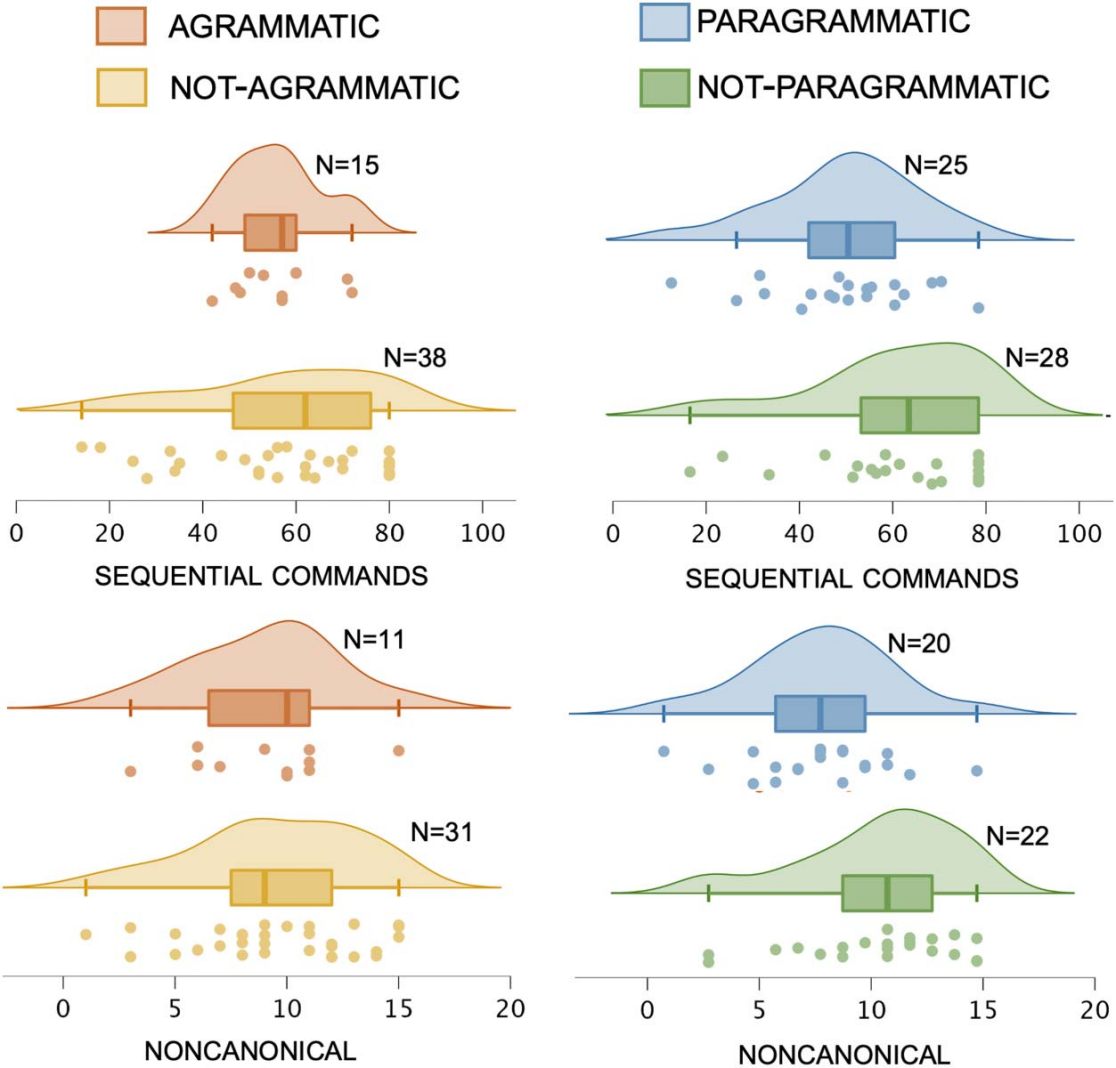
Behavioral performance on the two raw measures of syntactic comprehension (sequential commands and noncanonical), sorted by the categorical perceptual ratings of agrammatism and paragrammatism of Group 1 (total *N* = 53): agrammatic (*N* = 15) / not-agrammatic (*N* = 38), and paragrammatic (25) and not-paragrammatic (28). Dots indicate each individual data point within the relevant group, boxes and hash marks indicate a box-and-whisker plot with median and upper and lower quartiles, and curves represent estimated continuous distributions. (Top) sequential commands. (Bottom) noncanonical. The four participants classified as both agrammatic and paragrammatic were included in both groups for the analyses here.

**Figure 3.**
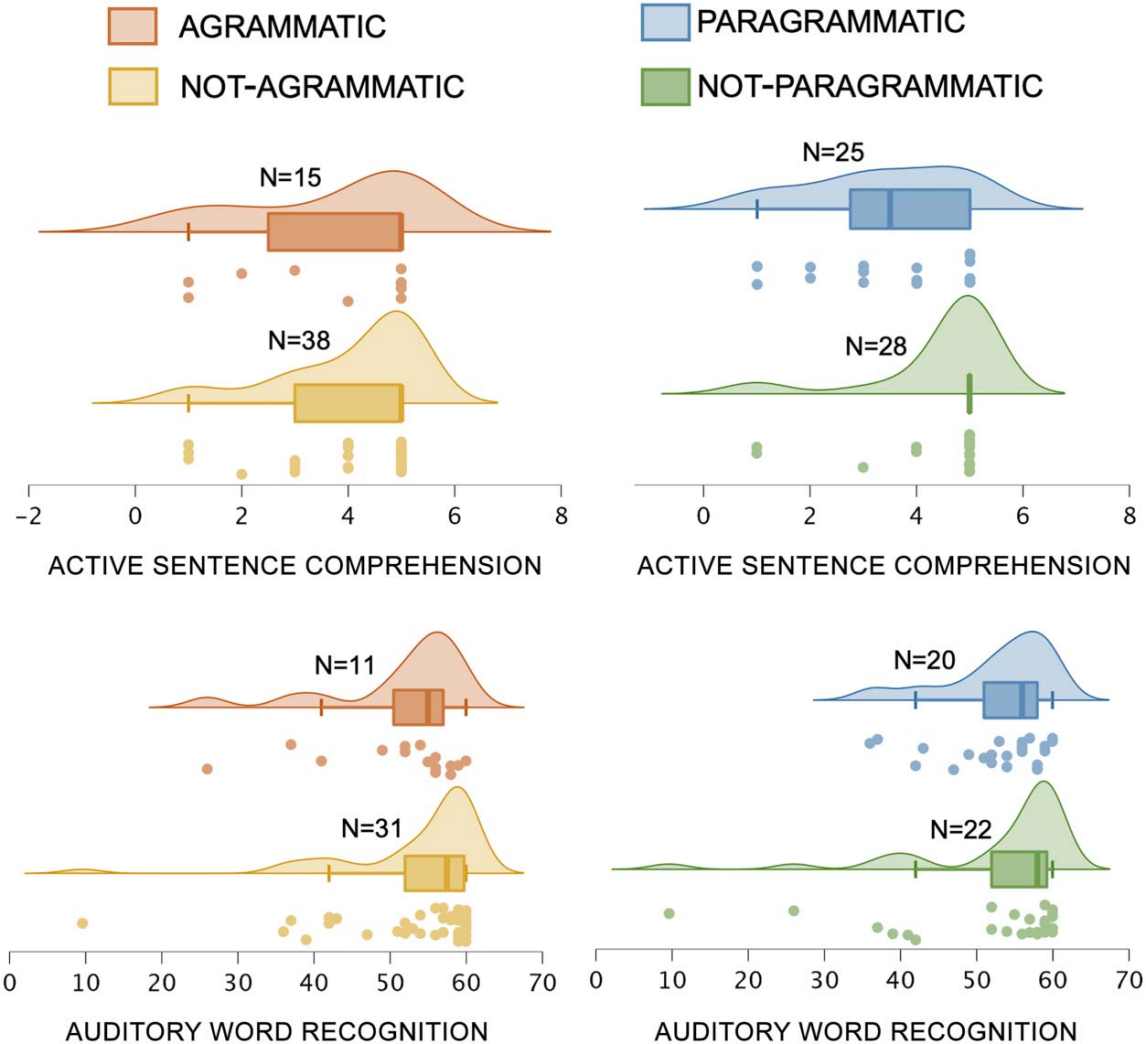
Behavioral performance on the two covariates (active sentence comprehension and auditory word recognition), sorted by the categorical perceptual ratings of agrammatism and paragrammatism of Group 1 (total *N* = 53): agrammatic (*N* = 15) / not-agrammatic (*N* = 38), and paragrammatic (*N* = 25) and not-paragrammatic (*N* = 28). Dots indicate each individual data point within the relevant group, boxes and hash marks indicate a box-and-whisker plot with median and upper and lower quartiles, and curves represent estimated continuous distributions. (Top) active sentence comprehension. (Bottom) auditory word recognition. The four participants classified as both agrammatic and paragrammatic were included in both groups for the analyses here.

**Figure 4.**
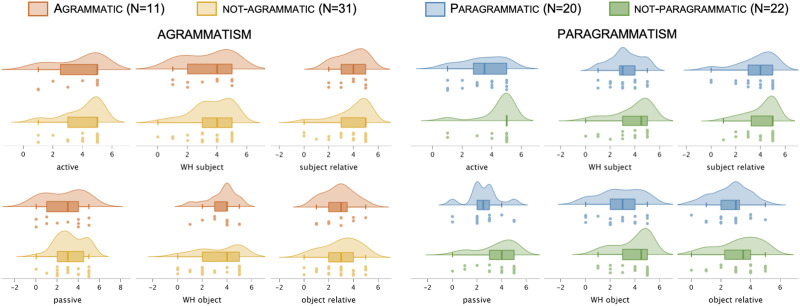
Behavioral performance on each individual canonical and noncanonical sentence type (*N* = 42), sorted by categorical perceptual ratings of agrammatism and paragrammatism. Dots indicate each individual data point within the relevant group, boxes and hash marks indicate a box-and-whisker plot with median and upper and lower quartiles, and curves represent estimated continuous distributions.

Figure 2 shows average performance in each group on the two primary syntactic comprehension measures (sequential commands and noncanonical), Figure 3 shows average performance in each group on the two behavioral covariates (auditory word recognition and active sentence comprehension), and Figure 4 shows average performance on all canonical and noncanonical sentence structures from the NAVS and ToS. Table 2 shows statistical results for all of the behavioral analyses involving these measures, *not* incorporating lesion volume as a covariate. There were no significant effects of agrammatism on performance for any of the syntactic comprehension measures. By contrast, there were significant effects of paragrammatism on performance for the analyses of sequential commands_audwords_ and noncanonical. Subtracting the significant effect sizes (partial *η*^2^) for these two analyses for agrammatism from paragrammatism revealed that the effect sizes were, following the guidelines of Cohen (1988), medium in strength greater for paragrammatism than agrammatism (0.082 and 0.133, respectively for the two behavioral measures). Supplementary analyses reported in Table S1 incorporating age, education, and WAB-R AQ as covariates showed similar results, albeit statistically weakened such that no analyses reached significance after correcting for multiple comparisons.

**Table 2.** Statistical results for behavioral analyses (multivariate linear regression) *without* incorporating lesion volume as a covariate.

	agrammatism	paragrammatism
sequential commands	*t* = −1.307, *p* = 0.197, partial *η*^2^ = 0.032	*t* = −1.459, *p* = 0.151, partial *η*^2^ = 0.040
sequential commands_audwords_	*t* = −1.217, *p* = 0.229, partial *η*^2^ = 0.029	*t = −2.501, *p* = 0.016, partial *η*^2^ = 0.111
noncanonical	*t* = −0.405, *p* = 0.688, partial *η*^2^ = 0.004	*t = −2.524, *p* = 0.016, partial *η*^2^ = 0.137
noncanonical_active_	*t* = −0.070, *p* = 0.945, partial *η*^2^ = 0.000	*t* = −1.305, *p* = 0.199, partial *η*^2^ = 0.042

*Note*. * = statistically significant result, using an adjusted alpha of *p* < 0.025, reflecting both the one-sided tests (negative associations only) and a Bonferroni correction for four multiple comparisons within each family of tests (agrammatism and paragrammatism considered as separate families).

### Behavioral Data, Incorporating Lesion Volume as a Covariate

We next performed four linear regression analyses examining the relationship between the scores for agrammatism and paragrammatism and syntactic comprehension, incorporating lesion volume as a covariate. Table 3 shows statistical results for each of these analyses. There were no significant effects of agrammatism on performance for any of the syntactic comprehension analyses. By contrast, there were significant effects of paragrammatism on performance for the analyses of sequential commands_audwords_ and noncanonical, and a trend toward significance for the analysis of sequential commands. Subtracting the significant effect sizes (partial *η*^2^) for these two analyses for agrammatism from paragrammatism revealed that the effect sizes were, following the guidelines of Cohen (1988), large in strength greater for paragrammatism relative to agrammatism (0.168 and 0.161, respectively for the two behavioral measures). Supplementary analyses reported in Table S2 incorporating age, education, and WAB-R AQ as covariates showed similar results, albeit statistically weakened such that no analyses reached significance after correcting for multiple comparisons.

**Table 3.** Statistical results for behavioral analyses *incorporating* lesion volume as a covariate (multivariate linear regression).

	agrammatism	paragrammatism
sequential commands	*t* = 0.632, *p* = 0.530, partial *η*^2^ = 0.008	*t* = −2.278, *p* = 0.027, partial *η*^2^ = 0.094
sequential commands_audwords_	*t* = −0.002, *p* = 0.999, partial *η*^2^ = 0	*t = −3.150, *p* = 0.003, partial *η*^2^ = 0.168
noncanonical	*t* = 0.205, *p* = 0.839, partial *η*^2^ = 0.001	*t = −2.721, *p* = 0.010, partial *η*^2^ = 0.160
noncanonical_active_	*t* = 0.700, *p* = 0.488, partial *η*^2^ = 0.013	*t* = −1.5, *p* = 0.142, partial *η*^2^ = 0.056

*Note*. * = statistically significant result, using an adjusted alpha of *p* < 0.025, reflecting both the one-sided tests (negative associations only) and a Bonferroni correction for four multiple comparisons within each family of tests (agrammatism and paragrammatism considered as separate families).

### Brain Lesion Data

**Figure 5.**
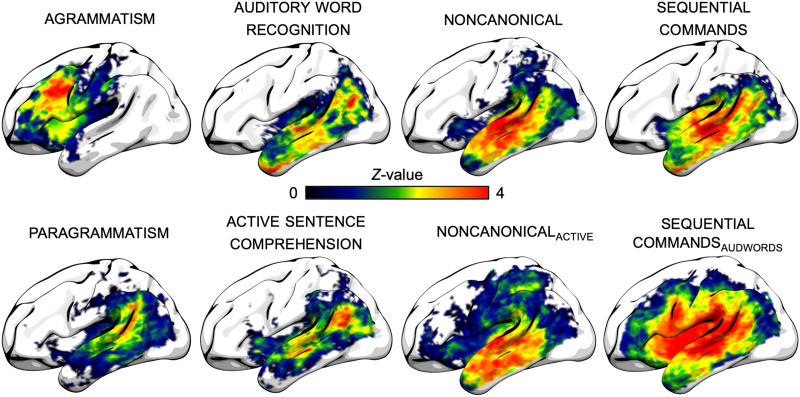
Whole-brain voxel-based analyses, unthresholded. All effects include lesion volume as a covariate.

Figure 5 shows unthresholded voxel-wise whole-brain lesion maps associated with each behavioral measure of interest. As reported in analyses of the same data set in Matchin et al. (2020), agrammatism (Group 1) was associated with damage primarily to inferior and middle frontal areas and anterior insula, and paragrammatism (Group 1) was associated with damage primarily to posterior superior temporal and inferior parietal lobe. As reported in analyses of the same data set in Matchin, Basilakos, et al. (2022), deficits in the lexical-semantic control measure auditory word recognition (Group 3) were associated with damage throughout the temporal and parietal lobes, but particularly the inferior portion of the angular gyrus, middle temporal lobe, and temporal pole. Deficits in the lexical-semantic control measure active sentence comprehension (Group 2) were associated with damage to the inferior portion of the angular gyrus and superior middle temporal lobe. As reported in analyses of a broader data set (including the proper subset of data reported here) in Matchin, den Ouden, et al. (2022), deficits in noncanonical (Group 2) were associated with damage throughout the temporal lobe, extending into the border of the inferior parietal lobe. As reported on analyses of the same data set in Matchin, Basilakos, et al. (2022), deficits in noncanonical after incorporating active sentence comprehension scores as a covariate, noncanonical_active_, (Group 2) were associated with damage throughout the temporal lobe. Deficits in sequential commands (Group 3, data not reported previously) were associated with somewhat similar patterns of damage to noncanonical, but extending into the temporal pole and the posterior insula. Deficits in sequential commands after incorporating auditory word recognition scores as a covariate, sequential commands_audwords_ (Group 3, data not reported previously), were associated with damage to the middle and posterior superior temporal lobe, posterior insula, and inferior precentral gyrus, extending into the inferior frontal gyrus.

We then determined the regions which were significantly associated with agrammatism and paragrammatism when correcting for multiple comparisons with permutation thresholding (10,000 permutations). agrammatism (with lesion volume as a covariate) was associated with damage to the posterior middle frontal gyrus (Z = 4.47), inferior frontal gyrus, pars opercularis (Z = 3.82), and inferior frontal gyrus, pars triangularis (Z = 3.47). paragrammatism (with lesion volume as a covariate) was associated with damage to posterior superior temporal gyrus (Z = 3.26), middle superior temporal gyrus (Z = 3.03), and posterior middle temporal gyrus (Z = 2.87).

We next examined the relationship between our measures of syntactic comprehension and percent damage to ROIs defined by the combined regions significantly associated with agrammatism (posterior middle frontal gyrus, inferior frontal gyrus, pars opercularis, and inferior frontal gyrus, pars triangularis combined) and paragrammatism (posterior superior temporal gyrus, middle temporal gyrus, and posterior middle temporal gyrus combined) using multivariate linear regression, incorporating lesion volume as a covariate in all analyses (Table 4). There were no significant relationships between residual damage to the agrammatism ROI on performance for any of the syntactic comprehension measures. By contrast, there were significant relationships between residual damage to the paragrammatism ROI on performance for all four syntactic comprehension analyses. Subtracting the significant effect sizes (partial *η*^2^) for these analyses for the agrammatism ROI from the paragrammatism ROI revealed that the effect sizes were, following the guidelines of Cohen (1988), medium to large in strength greater for the paragrammatism ROI relative to the agrammatism ROI (0.16, 0.108, 0.223, and 0.144, respectively for the four behavioral measures).

**Table 4.** Statistical results for lesion-deficit linear regression analyses between the brain regions implicated in grammatical production deficits and each measure of syntactic comprehension.

	agrammatism ROI	paragrammatism ROI
sequential commands	*t* = 2.129, *p* = 0.034, partial *η*^2^ = 0.021	*t = −5.896, *p* < 0.001, partial *η*^2^ = 0.139
sequential commands_audwords_	*t* = 0.054, *p* = 0.957, partial *η*^2^ = 0	*t = −5.081, *p* < 0.001, partial *η*^2^ = 0.108
noncanonical	*t* = 2.745, *p* = 0.007, partial *η*^2^ = 0.056	*t = −5.043, *p* < 0.001, partial *η*^2^ = 0.167
noncanonical_active_	*t* = 2.360, *p* = 0.020, partial *η*^2^ = 0.042	*t = −3.787, *p* < 0.001, partial *η*^2^ = 0.102

*Note*. * = statistically significant result, using an adjusted alpha of *p* < 0.025, reflecting both the one-sided tests (negative associations only) and a Bonferroni correction for four multiple comparisons within each family of tests (agrammatism and paragrammatism considered as separate families).

**Figure 6.**
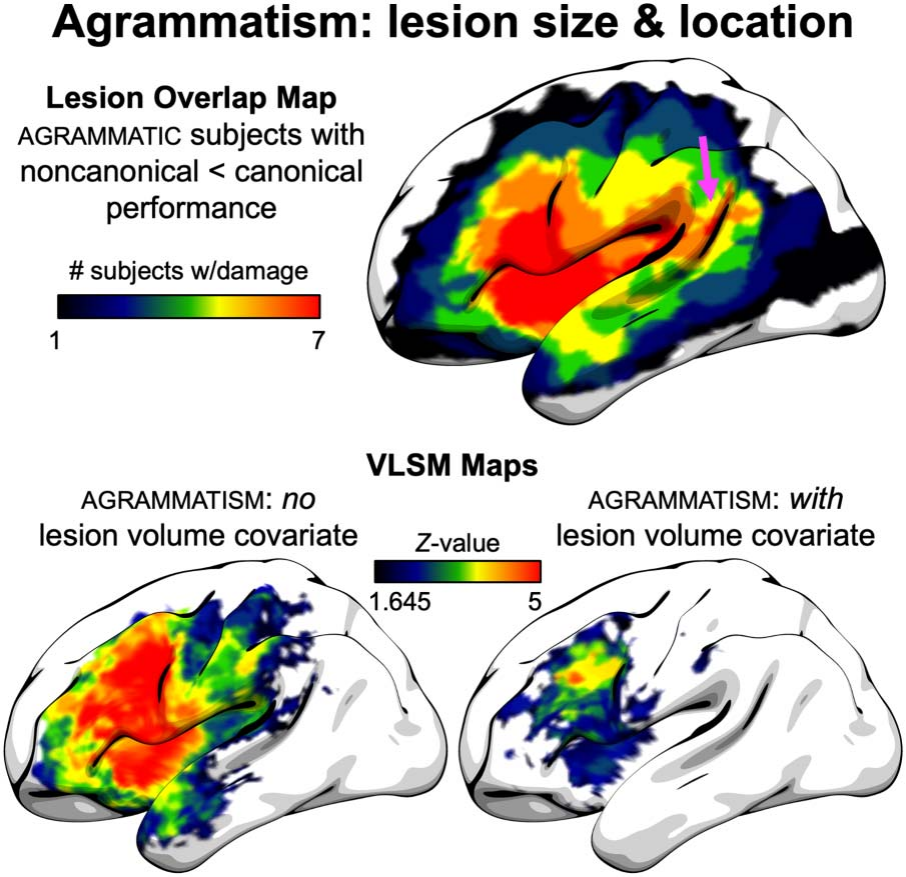
Agrammatism: lesion size and location. (Top) Lesion overlap map of subjects with expressive agrammatism and *agrammatic comprehension*, defined as worse performance on noncanonical relative to canonical structures. Arrow indicates area of maximum overlap in the posterior superior temporal sulcus. (Bottom) LSM analysis maps, thresholded at voxel-wise *p* < 0.05 (Z-value > 1.645), both without (left) and with (right) a lesion volume covariate.

Analyses regarding lesion volume and expressive agrammatism are shown in Figure 6. The lesion overlap map of agrammatic patients who also showed the classical pattern of agrammatic comprehension, worse performance on noncanonical relative to canonical structures, showed that all seven subjects with this profile had lesions in frontal and insular cortex, but there was also a region of maximum overlap in the posterior superior temporal lobe, including the posterior superior temporal sulcus. With respect to LSM analyses of expressive agrammatism, without including the lesion volume covariate, there were extensive effects throughout the frontal and parietal lobes, with significant anterior temporal damage and damage extending into posterior superior temporal gyrus and sulcus. When including the lesion volume covariate, the significant effects were almost entirely restricted to frontal lobe, with no temporal lobe involvement.

## DISCUSSION

### Reassessing Overarching Agrammatism

We found little evidence to support the classical overarching agrammatism hypothesis, the idea that parallel syntactic comprehension deficits coincide with expressive agrammatism (Caramazza & Zurif, 1976; Grodzinsky, 2000a; Schwartz et al., 1980; Thompson et al., 2013; Zurif, 1980). Given that patients with agrammatism and frontal damage had lesions much larger than those with paragrammatism or no grammatical deficit (Matchin et al., 2020), we suspected that previous studies finding associations between agrammatic Broca’s aphasia (with assumed damage to Broca’s area) and syntactic comprehension deficits, might be due in part to large lesions in these patients, possibly encroaching on the temporal lobe (see also Fridriksson et al., 2015). Our results support this account: With or without a lesion volume covariate, there were no significant associations between expressive agrammatism and syntactic comprehension deficits, and the two potentially suggestive trends present in the analyses without the lesion volume covariate completely disappeared when the lesion volume covariate was included. Furthermore, including a lesion volume covariate, damage to regions associated with agrammatism (Broca’s area and posterior middle frontal gyrus) was not associated with any of the syntactic comprehension measures. Overall, the data cast strong doubt on the overarching agrammatism hypothesis.

The lack of any association in our data between expressive agrammatism and deficits in noncanonical sentence comprehension seemingly contradicts previous studies (Caramazza & Zurif, 1976; Cho-Reyes & Thompson, 2012; Schwartz et al., 1980; Thompson et al., 2013), but it is important to note that this does not mean that our agrammatic patients had no deficits in comprehending these sentence structures. Rather, it illustrates that agrammatism itself, out of a group of people with aphasia, is not particularly strongly associated with such deficits; that is, people with aphasia but not agrammatism were not significantly different in their ability to comprehend noncanonical sentence structures. This underscores the importance of an appropriate comparison group; some previous studies only examined agrammatic patients (Caplan & Futter, 1986; Grodzinsky, 2000b; Schwartz et al., 1980) or compared agrammatic patients to those with mild anomic aphasia (Cho-Reyes & Thompson, 2012), which conflates expressive agrammatism with overall aphasia severity and/or lesion volume.

Note that our results do not imply that inferior frontal cortex regions never play any role in sentence comprehension. In particular, frontal-motor systems are widely thought to play a key role in working memory (Baddeley, 2003; Baddeley et al., 1981; Pettigrew & Hillis, 2014; Rogalsky et al., 2008). Working memory is important for sentence comprehension, particularly for difficult constructions and perhaps other demanding aspects of everyday natural communication (being presented with false starts and stops, garden-paths/misparsing, preparing to respond to the interlocuter). In particular, the linear morpho-syntactic system supported by the pars triangularis of Broca’s area, as posited by Matchin and Hickok (2020), is ideal for assisting comprehension by reiterating the heard sequence of morphemes for reanalysis by hierarchical syntactic mechanisms in the posterior temporal lobe. This is congruent with the hypothesis of a syntactic working memory system (Fiebach et al., 2005; Matchin, 2018; Rogalsky et al., 2015) consisting of looping interactions between inferior frontal cortex (typically used for linear sequencing during production) and posterior temporal cortex. As such, a working memory deficit may help explain some of the reported associations between agrammatic Broca’s aphasia and deficits in the comprehension of semantically reversible, complex, noncanonical sentences (although we note that a recent LSM study assessed this possibility and did not find any support for it; Rogalsky et al., 2018). Furthermore, working memory deficits (along with deficits in visual-motor processing) might account for the fact that agrammatism was very weakly (and nonsignificantly) associated with deficits in sequential commands and sequential commands_audwords_ when lesion volume was not taken into account, given that the Sequential Commands subtest of the WAB-R requires participants to keep a series of actions in memory to correctly perform more complex items.

A role for frontal-motor systems in syntactic working memory may also explain some of the syntax-related activations that are observed in inferior frontal cortex in neuroimaging studies (Rogalsky & Hickok, 2011). While early studies found evidence for syntactic comprehension effects primarily in Broca’s area, recent neuroimaging studies have found that posterior temporal cortex shows equally reliable syntactic comprehension effects (Blank et al., 2016; Diachek et al., 2020; Fedorenko et al., 2012, 2016; Goucha & Friederici, 2015; Matchin et al., 2017; Meyer & Friederici, 2016; Nelson et al., 2017; Pallier et al., 2011; Shain et al., 2020; Zaccarella, Meyer, et al., 2017; Zaccarella, Schell, et al., 2017); in fact, some studies have shown that posterior temporal lobe shows an activation profile more consistent with fuller aspects of abstract hierarchical structure building than frontal cortex (Brennan, 2016; Fedorenko et al., 2020). The fact that both of these regions reliably exhibit syntactic effects is well-explained by attributing a syntactic function to both of these regions (Matchin & Hickok, 2020), with the frontal contribution reflecting production-related morpho-syntactic resources that assist comprehension in demanding contexts, but that are not necessary for combining words into structured phrases and sentences, a role reserved for the posterior temporal lobe.

Some LSM studies have found an association between damage and/or degeneration of inferior frontal cortex and deficits in comprehension of complex, noncanonical sentence structure, typically in addition to posterior temporal damage (Adezati et al., 2022; Amici et al., 2007; Fridriksson et al., 2018; Kristinsson et al., 2020; Magnusdottir et al., 2013; Mesulam et al., 2015; Tyler et al., 2011; Wilson et al., 2011). However, there are several reasons to question whether these results reflect necessary resources for normal syntactic comprehension.

First, most of these studies did not incorporate lesion volume as a covariate, which is an important variable to ensure accurate localization in LSM (DeMarco & Turkeltaub, 2018; Ivanova et al., 2021). This is a particularly acute issue given that agrammatic production is associated with large lesions (Matchin et al., 2020) that encroach on the temporal lobe (Fridriksson et al., 2015). Our lesion overlap analysis showed that 100% of agrammatic subjects who also showed the traditional agrammatic comprehension profile (worse performance on noncanonical relative to canonical sentences) had lesions extending into the posterior temporal lobe. It is possible that syntactic comprehension deficits in agrammatic patients in older studies may have been due to temporal lobe damage rather than the frontal lobe lesions that were often assumed.

Second, many LSM studies have reported no association between syntactic comprehension and frontal lobe structures even for complex structures (den Ouden et al., 2019; Dronkers et al., 2004; Matchin, Basilakos, et al., 2022; Matchin, den Ouden, et al., 2022; Rogalsky et al., 2018; Thothathiri et al., 2012). Third, complex noncanonical structures critically involve substantial working memory resources (King & Just, 1991; Pettigrew & Hillis, 2014; Rogalsky et al., 2008), which could be the source of the associations with frontal networks as noted. Fourth, LSM of sentence comprehension in general does not highlight Broca’s area, but rather posterior temporal and inferior parietal cortex, similar to the areas we identified here (Baldo & Dronkers, 2007; Dronkers et al., 2004; Fridriksson et al., 2018; Pillay et al., 2017; Rogalsky et al., 2018; Thothathiri et al., 2012), and most sentences, regardless of their complexity and canonicity, likely draw on at least some syntactic resources in order to establish the basic thematic relations of sentences. Finally, LSM studies have shown that damage to posterior temporal lobe, but not Broca’s area, is associated with syntactic acceptability judgment deficits (Fahey et al., 2021; Wilson & Saygın, 2004). However, these studies have limited numbers of participants. Large-scale LSM studies using a variety of alternative measures of syntactic comprehension ability, including acceptability judgments, are needed to more firmly assess the extent to which the posterior temporal lobe is engaged in receptive syntactic processing that cannot be accounted for by individual word-level conceptual-semantic and/or phonological deficits.

Interestingly, a recent cortical stimulation study in six participants did find that stimulation of Broca’s area (primarily pars opercularis) resulted in deficits in comprehension of passive (but not active) sentences (Riva et al., 2022), which aligns with a case study of acute stroke resulting in relatively focal hypoperfusion of Broca’s area with impaired syntactic comprehension (Davis et al., 2008). This is consistent with the possibility that Broca’s area plays a supporting role that, when acutely disrupted, results in dysfunction that resolves over time, whereas posterior temporal lobe plays a fundamental syntactic structure building function.

### Paragrammatism, Syntactic Comprehension Deficits and the Posterior Temporal Lobe

By contrast to our investigation of the relationship between expressive agrammatism and syntactic comprehension deficits, both our behavioral measure of paragrammatism, and damage to the paragrammatism ROI (middle and posterior superior temporal gyrus and posterior middle temporal gyrus) were significantly associated with some syntactic comprehension deficits, whether lesion volume was included as a covariate or not. These results suggest that while both posterior temporal and frontal cortex play important roles in syntax, they do so asymmetrically: Posterior temporal cortex is critically involved in both comprehension and production, whereas frontal cortex is only critically involved in production. A functional-anatomical asymmetry is supported by recent fMRI studies which have found that these regions diverge accordingly with respect to syntactic measures in production and comprehension (Giglio et al., 2022; Matchin & Wood, 2020). This is consistent with previous functional-anatomical asymmetries identified in the phonological domain (Hickok & Poeppel, 2004).

These results suggest the possibility of a syntactic parallelism hypothesis rooted in paragrammatism rather than agrammatism. Roots of this idea extend back to Wernicke (1874), who argued that the temporal lobe subserved both receptive and expressive function at the speech-sound level. These ideas were adapted to syntax in the framework advanced by Matchin and Hickok (2020): The posterior temporal lobe (crucially including ventral superior temporal sulcus) underlies a hierarchical lexical syntactic function for interfacing with brain systems involved in processing meaning, whereas the pars triangularis of Broca’s area and perhaps a more dorsal region in the middle frontal gyrus underlies a morpho-syntactic sequencing function that interfaces with the motor system. Thus, it is possible that the more abstract idea underlying overarching agrammatism is viable—that a central syntactic system can be localized to particular part of the brain and supports both production and comprehension, but in the posterior temporal lobe and not the frontal lobe.

### Limitations

We focused on the question of whether or not an overarching agrammatism hypothesis is supported, in accordance with traditional claims in the literature, and whether or not an alternative overarching paragrammatism hypothesis is supported. We are not claiming that the significant effects we identified in paragrammatism are *stronger* than those in agrammatism, only that we can identify an association between grammatical production and comprehension deficits only for paragrammatism, and not for agrammatism. Importantly, while we did find evidence of a relationship between paragrammatism and deficits in some syntactic comprehension measures, this was not overwhelmingly robust, and some of the measures did not reach significance. We did not have handedness or education information for all subjects, which are important variables that are known to relate to aphasia outcome (Lwi et al., 2021). Importantly, while we did find evidence of a relationship between paragrammatism and deficits in some syntactic comprehension measures, this was not overwhelmingly robust, and some of the measures did not reach significance (and the statistical results were weaker when incorporating age, education, and WAB-R AQ as covariates; see Supplementary Data).

Additionally, while there was a statistically strong relationship between damage to the regions implicated in paragrammatism and syntactic comprehension deficits, the whole-brain lesion maps for these measures showed some important differences in the lesion distributions for these measures. The sequential commands and sequential commands_audwords_ analyses, while designed to avert some of the limitations of working memory demands required of complex structures, likely still involved some degree of phonological working memory resources which shifted the lesion distribution superiorly away from the middle temporal gyrus. Likewise, the analyses of noncanonical and noncanical_active_ likely taxed working memory resources and/or conceptual-semantic processing in addition to syntax. In addition, the speech of patients with fluent aphasia, who are overrepresented in the paragrammatic group analyzed here relative to the agrammatic group, frequently contains phonological and semantic errors, some of which may contribute to the overall impression of paragrammatic speech output used for categorization in this study. New reliable and valid measures of agrammatic and paragrammatic speech production and syntactic comprehension abilities are needed to more clearly investigate these issues.

Finally, although LSM provides important insights into the organization of the brain, complementary to functional neuroimaging in healthy subjects, it also has complicating factors. For example, patterns of functional disruption due to stroke may be complex and difficult to assess purely from the standpoint of lesions visually identifiable on MRI scans. In addition, particularly in chronic stroke there may be functional reorganization, which makes interpretation of lesion-deficit correlation results difficult to interpret. Thus, we continue to advocate for the combination of methods, including both LSM and functional neuroimaging (and other methods, such as brain stimulation), to provide the fullest insights into the organization of syntax in the brain.

## CONCLUSIONS

In this work, we would like to reinforce two main points. First, behaviorally, paragrammatism, and not expressive agrammatism, is associated with behavioral deficits in syntactic comprehension, when assessed from a variety of perspectives and covariates. Second, neurologically, there is convergence in the brain lesions associated with syntactic comprehension and paragrammatism but not agrammatism. Crucially, our results may conflict with previous studies because we included a full range of patient types, rather than solely comparing agrammatic patients to people without aphasia or only mild anomic aphasia (cf. Thompson et al., 2013, which examined both stroke-based aphasia and primary progressive aphasia, comparing agrammatic patients to those with significantly less severe aphasia), and investigated the role of lesion size. This provides strong evidence against the concept of “overarching agrammatism” rooted in the frontal lobe, and provides some converging evidence for a role for posterior temporal lobe in a central syntactic processing mechanism that supports both comprehension and production.

## ACKNOWLEDGMENTS

We wish to thank Leonardo Bonilha, Roger Newman-Norlund, and Chris Rorden for their assistance.

## FUNDING INFORMATION

Alexandra Basilakos, National Institute on Deafness and Other Communication Disorders (https://dx.doi.org/10.13039/100000055), Award ID: DC014435. Julius Fridriksson, National Institute on Deafness and Other Communication Disorders (https://dx.doi.org/10.13039/100000055), Award ID: DC014664. Julius Fridriksson, National Institute on Deafness and Other Communication Disorders (https://dx.doi.org/10.13039/100000055), Award ID: DC011739.

## AUTHOR CONTRIBUTIONS

**William Matchin**: Conceptualization; Formal analysis; Investigation; Project administration; Visualization; Writing – original draft; Writing – review and editing. **Dirk-Bart den Ouden**: Investigation; Project administration; Writing – review and editing. **Alexandra Basilakos**: Data curation; Investigation; Writing – review and editing. **Brielle Caserta Stark**: Investigation; Writing – review and editing. **Julius Fridriksson**: Funding acquisition; Resources. **Gregory Hickok**: Conceptualization; Writing – review and editing.

## DATA AVAILABILITY STATEMENT

All data are publicly available using the following link (use the grammaticalParallelism_data_8.3.2022.xlsx spreadsheet): https://www.dropbox.com/home/Matchin_publiclyShared_C-STAR_lesionData.

## TECHNICAL TERMS

Syntax: The structure of sentences that supports combinatorial and creative use of language.
Agrammatism: A language production disorder involving the omission of functional elements, the overall simplification of sentence structure, and telegraphic speech.
Paragrammatism: A language production disorder involving the misuse of functional elements and structures, without an overall tendency toward omission or reduction.
Lesion-symptom mapping: A statistical technique that allows researchers to associate patterns of brain damage with behavioral performance.
Sequential Commands: A subtest of the WAB-R that involves asking the participant to perform increasingly complex actions that require comprehending sentence structure.
Western Aphasia Battery—Revised (WAB-R): A standard battery of language comprehension, production, and repetition abilities; supports aphasia classification and severity assessment.
Auditory Word Recognition: A subtest of the WAB-R that involves asking the participant to identify common everyday objects after hearing their labels.
Noncanonical sentence comprehension: A standard metric of syntactic comprehension abilities, involving sentences with different word order than is typically expected within the language.
Magnetic resonance imaging (MRI): A neuroimaging technique that allows for precision mapping of both brain structure and brain activity.
