# Supplementary material for: Grammatical Parallelism in Aphasia: A Lesion-Symptom Mapping Study
Source: Neurobiol Lang (Camb). 2023 Oct 31;4(4):550–74. doi: 10.1162/nol_a_00117 (PMC10631800; doi:10.1162/nol_a_00117)
Supplement: Supplementary file 1 [file nol-4-4-550-s001.pdf]

## Supplementary Data

Here we report supplementary behavioral analyses adding age at testing, years of education, and WAB-R AQ as additional covariates. N.B.: we did not have years of education information for all participants, as detailed in Table 1. First, we performed two linear regressions on both of the primary measures of syntactic comprehension (SEQUENTIAL COMMANDS and NONCANONICAL), one including age at testing, years of education, and WAB-R AQ as covariates, and one adding the lexical-semantic controls as an additional covariate (WAB-R AUDITORY WORD RECOGNITION for $\text{SEQUENTIAL COMMANDS}_{\text{AUDWORDS}}$, and ACTIVE SENTENCE COMPREHENSION for $\text{NONCANONICAL}_{\text{ACTIVE}}$). Then, in the identical fashion as reported in the main text, we performed two sets of analyses on these four resulting behavioral measures, one without incorporating lesion volume as a covariate (Table S1) and one incorporating lesion volume as a covariate (Table S2).

The results overall were similar to those reported in the main text without including these additional covariates: there were no significant effects for the relationship between agrammatism and any syntactic comprehension measures, whether lesion volume was included or not. The behavioral analyses for paragrammatism were substantially weakened, with no analyses passing the threshold for significance, although the analysis of $\text{SEQUENTIAL COMMANDS}_{\text{AUDWORDS}}$ incorporating the lesion volume covariate neared significance.

| | AGRAMMATISM | PARAGRAMMATISM |
|---|---|---|
| SEQUENTIAL COMMANDS | t = -0.126, *p* = 0.901 | t = -0.445, *p* = 0.659 |
| $\text{SEQUENTIAL COMMANDS}_{\text{AUDWORDS}}$ | t = -0.352, *p* = 0.727 | t = -1.868, *p* = 0.068 |
| NONCANONICAL | t = -0.434, *p* = 0.667 | t = -1.828, *p* = 0.076 |
| $\text{NONCANONICAL}_{\text{ACTIVE}}$ | t = -0.253, *p* = 0.802 | t = -0.927, *p* = 0.360 |

Table S1. Statistical results for behavioral analyses *without* incorporating lesion volume as a covariate, but with covariates for age at testing, level of education, and WAB-R AQ. We used an adjusted alpha of $p < 0.025$ to determine significance, reflecting both the one-sided tests (negative associations only) and a Bonferroni correction for four multiple comparisons within each family of tests (AGRAMMATISM and PARAGRAMMATISM considered as separate families), but no results surpassed this threshold.

| | AGRAMMATISM | PARAGRAMMATISM |
|---|---|---|
| SEQUENTIAL COMMANDS | t = 0.259, *p* = 0.797 | t = -0.686, *p* = 0.496 |
| $\text{SEQUENTIAL COMMANDS}_{\text{AUDWORDS}}$ | t = -0.027, *p* = 0.978 | t = -2.206, *p* = 0.032 |
| NONCANONICAL | t = -0.411, *p* = 0.684 | t = -1.923, *p* = 0.062 |
| $\text{NONCANONICAL}_{\text{ACTIVE}}$ | t = 0.094, *p* = 0.926 | t = -1.088, *p* = 0.284 |

Table S2. Statistical results for behavioral analyses *incorporating* lesion volume as a covariate, as well as age at testing, level of education, and WAB-R AQ. We used an adjusted alpha of $p < 0.025$ to determine significance, reflecting both the one-sided tests (negative associations only) and a Bonferroni correction for four multiple comparisons within each family of tests (AGRAMMATISM and PARAGRAMMATISM considered as separate families), but no results surpassed this threshold.
